# Editorial: NSAIDs Pharmacogenomics

**DOI:** 10.3389/fphar.2021.798447

**Published:** 2021-11-19

**Authors:** José A. G. Agúndez, Christine Formea, Andrea Gaedigk, Elena García-Martín, Li Gong, Tilo Grosser, Craig R. Lee, Katherine N. Theken

**Affiliations:** ^1^ Universidad de Extremadura, Institute of Molecular Pathology Biomarkers, University of Extremadura, Cáceres, Spain; ^2^ Intermountain Healthcare, Salt Lake City, UT, United States; ^3^ Children’s Mercy, Kansas City, MO, United States; ^4^ Stanford University, Stanford, CA, United States; ^5^ University of Pennsylvania, Philadelphia, PA, United States; ^6^ University of North Carolina at Chapel Hill, Chapel Hill, NC, United States

**Keywords:** pharmacogenomics (PGx), non-steroidal anti-inflammatory drug (NSAID), pharmacodynamic (PD), pharmacokinectics & drug metabolism (PDM), adverse drug effects

The research topic “NSAIDs Pharmacogenomics” comprises eight articles to which more than 60 authors contributed. The main aim of this research topic was to gather a collection of articles that would fill relevant gaps on the influence of genetic variation in genes involved in the pharmacokinetics of NSAIDs, drug response, or the risk of developing NSAID-related adverse events.

Major NSAID-related adverse events continue to pose a profound challenge for individual and public health. Major adverse events affect the gastrointestinal, renal, or cardiovascular systems, and include hypersensitivity reactions that, on occasion, can be life-threatening. NSAID-related adverse events are common, accounting for perhaps up to 30% of hospitalizations due to adverse drug reactions (Pirmohamed et al., 2004).

Despite the extensive work carried out in this field that has led, for instance, to the development of the Clinical Pharmacogenetics Implementation Consortium (CPIC) guideline for *CYP2C9* and NSAIDs (Theken et al., 2020). However, there are still many knowledge gaps including the potential contribution of other CYP and non-CYP genes to the metabolism of NSAIDs, drug targets affecting response, as well as the pharmacokinetics, therapeutic effects, and the risk of developing adverse events with NSAIDs and other COX inhibitors.

Two articles in this research topic addressed gastrointestinal bleeding which is one of the most relevant and life-threatening adverse effects of NSAIDs. McEvoy et al. reviewed genetic factors involved in NSAID-induced gastrointestinal toxicity and concluded that CYP2C genetic variants are neither necessary nor sufficient to predispose to NSAID-induced peptic ulcer disease. However, low CYP2C9 activity may increase gastrointestinal toxicity risk after the use of NSAIDs that are metabolized by CYP2C9. The authors also highlight the potential role of the interaction between genetic factors and multimorbidity and polypharmacy. Mallah et al. studied the contribution of gene variation to upper gastrointestinal hemorrhage in aspirin users. In contrast to other NSAIDs, CYP metabolism only plays a minor role in aspirin biodisposition (Agundez et al., 2009) and no strong genetic associations with aspirin-related gastrointestinal events have been described so far. The authors analyzed 25 single nucleotide variants (SNVs) in genes involved in platelet activation, angiogenesis, and inflammatory response. The strongest positive associations were related to three intronic (SNVs) and therefore, these preliminary findings require further studies with larger sample sizes and additional gene expression analyses.

Another major NSAID-related adverse event is hypersensitivity. Approximately two to four percent of adults have reported at least one event of hypersensitivity to NSAIDs (Gomes et al., 2004; Zhou et al., 2016). This, together with the wide consumption of these drugs without a prescription makes NSAID hypersensitivity a major health challenge. Trinh et al. reviewed recent advances in NSAID hypersensitivity, emphasizing the role of the arachidonic acid pathway in the development of these reactions, the genetic variants involved in NSAID hypersensitivity, and the role of epigenetics. The authors conclude that the various clinical phenotypes and subphenotypes observed in NSAID hypersensitivity are the consequence of mechanisms regulated by genetic and epigenetic variants and possible interactions between them, which could manifest differently among populations. Jurado-Escobar et al. analyzed the role of genetic variation in the cytosolic phospholipase A_2_ gene (*PLA2G4A*), which is crucial in the bioavailability of arachidonic acid. They identified three SNVs that were related to the risk of developing NSAID-induced acute urticaria and angioedema. This study adds to the potential role of variations in the genes encoding drug targets, that is COX-1 and COX-2, in the development of adverse events with NSAIDs (Garcia-Martin et al., 2021). Macías et al. analyzed the potential impact of polymorphic CYP2C-mediated NSAID metabolism in the risk of developing cross-hypersensitivity to NSAIDs. The fact that cross-hypersensitivity is linked to COX-inhibition, supports the hypothesis that individuals with impaired NSAID biodisposition might be at an increased risk of developing this adverse drug reaction. Common functional variants in *CYP2C* genes were analyzed in a large cohort. The only statistically significant association identified was restricted to NSAIDs that are CYP2C8/9 substrates. Individuals who are homozygous for the CYP2C8*3 alleles appeared to exhibit an increased risk of developing cross-hypersensitivity. However, this association was not statistically significant after accounting for multiple comparisons, and the study concluded that there is no evidence implicating the most common functional CYP2C polymorphisms and the risk of developing cross-hypersensitivity to NSAIDs.

Finally, two articles analyzed the role of genetic variation impacting NSAID pharmacokinetics and pharmacodynamic effects. Mejía-Abril et al. investigated the role of several functional variations in the genes *CYP1A2*, *CYP2A6*, *CYP2B6*, *CYP2C8*, *CYP2C9*, *CYP2C19*, *CYP2D6*, *CYP3A4*, *CYP3A5*, *ABCB1*, *ABCC2*, *SLCO1B1*, *SLC22A1*, and *UGT1A1* on the pharmacokinetics of dexketoprofen. Although the number of participants was small, the authors identified an association between a synonymous *ABCB1* variant, as well as weak associations with *CYP1A2*, *CYP2B6*, and *CYP2D6* and dexketoprofen exposure. Overall, no major associations with the pharmacokinetics of dexketoprofen were identified, however. Weckwerth et al. studied the role of common variants in the OPRM1 and COMT genes, as well as interleukin and interferon concentrations in saliva, on postoperative pain in 200 individuals who received 600 ibuprofen. The study is based on the hypothesis that polymorphisms in OPRM1 and COMT would impact β-endorphin and catecholaminergic signaling, respectively, which may result in differences in perception and modulation of pain and analgesia. The study concluded that, among other factors, variants in the COMT gene, and the concentrations of IL-2 and IFN-γ in saliva before surgery were predictors for pain perception. While these findings warrant replication, they may add to the well-established role of genetic variation in ibuprofen biodisposition (and hence effects), thus identifying additional factors that could impact analgesic efficacy.

The collection of articles included in the research topic “NSAIDs Pharmacogenomics” highlights the importance of deep phenotyping in studies addressing variation in drug action, since the mechanisms underlying NSAID response seem to be multifactorial and phenotype-dependent. The genetics of the response to NSAIDs are more complex than one might expect for a drug class as commonly consumed as this one, and the underlying mechanisms and risk factors for NSAID-related adverse events appear to differ in patients with different subphenotypes. Although the articles included in this research topic shed light on key factors of NSAID pharmacogenomics, additional research is needed to gain further insights as we move toward improving the safety and efficacy of NSAIDs by precision therapy.
